# Historical Distribution and Molecular Diversity of *Bacillus anthracis*, Kazakhstan

**DOI:** 10.3201/eid1605.091427

**Published:** 2010-05

**Authors:** Alim M. Aikembayev, Larissa Lukhnova, Gulnara Temiraliyeva, Tatyana Meka-Mechenko, Yerlan Pazylov, Sarkis Zakaryan, Georgiy Denissov, W. Ryan Easterday, Matthew N. Van Ert, Paul Keim, Stephen C. Francesconi, Jason K. Blackburn, Martin Hugh-Jones, Ted Hadfield

**Affiliations:** Kazakhstan Scientific Center for Quarantine and Zoonotic Diseases, Almaty, Kazakhstan (A.M. Aikembayev, L. Lukhnova, G. Temiraliyeva, T. Meka-Mechenko, Y. Pazylov, S. Zakaryan, G. Denissov); Northern Arizona University, Flagstaff, Arizona, USA (W.R. Easterday, P. Keim); Midwest Research Institute, Palm Bay, Florida, USA (M.N. Van Ert, T. Hadfield); The Translational Genomics Research Institute, Phoenix, Arizona, USA (P. Keim); Naval Medical Research Center, Silver Spring, Maryland, USA (S.C. Francesconi); California State University, Fullerton, California, USA (J.K. Blackburn); Louisiana State University, Baton Rouge, Louisiana, USA (M. Hugh-Jones); 1 Current affiliation: Republican Sanitary Epidemiologic Station, Almaty, Kasakhstan.; 2 Current affiliation: VEN Consulting, LLC, Melbourne, Florida, USA.; 3 Current affiliation: University of Florida, Gainesville, Florida, USA.

**Keywords:** Bacillus anthracis, anthrax, genotyping, MLVA, SNP, GIS, spatial analysis, bacteria, research

## Abstract

To map the distribution of anthrax outbreaks and strain subtypes in Kazakhstan during 1937–2005, we combined geographic information system technology and genetic analysis by using archived cultures and data. Biochemical and genetic tests confirmed the identity of 93 archived cultures in the Kazakhstan National Culture Collection as *Bacillus anthracis*. Multilocus variable number tandem repeat analysis genotyping identified 12 genotypes. Cluster analysis comparing these genotypes with previously published genotypes indicated that most (n = 78) isolates belonged to the previously described A1.a genetic cluster, 6 isolates belonged to the A3.b cluster, and 2 belonged to the A4 cluster. Two genotypes in the collection appeared to represent novel genetic sublineages; 1 of these isolates was from Krygystan. Our data provide a description of the historical, geographic, and genetic diversity of *B. anthracis* in this Central Asian region.

## Abstract 2

This study provides useful baseline data for guiding future disease control programs.

Anthrax is a globally widespread disease of livestock and wildlife that occasionally infects humans. According to official estimates, the number of human anthrax cases worldwide ranges from 2,000 to 20,000 annually (*1*)*. Bacillus anthracis*, the etiologic agent of anthrax, persists in the environment as a dormant, highly stable spore. The prolonged periods of dormancy during the spore phase slows evolution of this species, resulting in high levels of interstrain genetic homogeneity, which complicates efforts to subtype the pathogen. The availability of whole-genome nucleotide sequences of *B. anthracis* for single-nucleotide polymorphism (SNP) elucidation and the discovery of polymorphic markers such as variable number tandem repeat (VNTR) sequences (*2**,**3*) have enabled identification of unique subtypes within this species. Keim et al. (*4*) used 8 VNTRs to describe 89 unique genotypes in a global collection of over 400 *B. anthracis* isolates. Later studies used VNTRs to examine *B. anthracis* diversity in different global regions, including France (*5*)*,* Italy (*6*)*,* Poland (*7*)*,* Chad (*8*), and South Africa (*9*)*.* More recently, SNPs that define major clonal lineages in *B. anthracis* have been identified and applied to describe global and regional patterns of *B. anthracis* diversity (*10*)*.*

In the central Asian republic of Kazakhstan, anthrax is enzootic and still represents a human public health concern. A recent publication examined risk factors associated with 73 human anthrax cases in Kazakhstan over a 2-year period (*11*) and concluded that most cases were cutaneous and had resulted from the handling of infected livestock and contaminated animal products. Gastrointestinal anthrax in Kazakhstan has also been reported but is less common. Despite the widespread nature of the disease in this region, the historical incidence, distribution, and genetic diversity of *B. anthracis* in central Asia, and Kazakhstan in particular, has remained cryptic.

We mapped the historical distribution of anthrax in Kazakhstan over a 68-year period. Archived cultures from a subset of these outbreaks collected from 10 oblasts (provinces) over a 53-year period were analyzed by using genetic and biochemical tests. Multilocus variable number tandem repeat analysis (MLVA) and canonical single nucleotide polymorphism genotyping (*10*) of this collection enabled us to examine strain dynamics among and within these outbreaks and to understand the diversity of *B. anthracis* isolates from Kazakhstan on a local, regional, and global scale.

## Materials and Methods

### Mapping Historical Anthrax Outbreaks

To map the historical distribution of anthrax outbreaks and *B. anthracis* strain types across Kazakhstan, we constructed a geographic information system (GIS) database within ArcGIS 9.1 (www.esri.com). This database used archival data collected through the antiplague stations established by the Union of Soviet Socialist Republics. This system of stations remains in place under the current government, and Kazakhstan maintains a multiagency reporting protocol to update, document, and respond to the distribution of outbreaks. These data are archived at the Kazakhstan Scientific Center for Quarantine and Zoonotic Diseases. Outbreaks and strain locations were geolocated to the nearest village by using GIS data layers produced by the Kazakh Institute of Geography. Historical outbreaks were mapped for 1937 through 2005. To illustrate differences in the distributions of outbreaks in cattle and sheep, the 2 most affected livestock species, a kernel density estimation was performed by using the Spatial Analyst Extension in ArcGIS. We mapped outputs by using the standard deviation of density values to illustrate areas of greatest outbreak concentration by species (*12*)*.*

### Isolation of *B. anthracis*

Samples collected from anthrax outbreaks in Kazakhstan (with the exception of 2 isolates from the Kyrgyzstan border region) and cultures spanning a 53-year period were archived in the Kazakhstan National *B. anthracis* Collection. Most isolates were from human patients, some from blood or organs of ruminants (mainly sheep and cows), and a few from soil or other inanimate objects contaminated by contact with blood or tissues of infected animals. Archived cultures were confirmed as *B. anthracis* on the basis of colony morphologic appearance; absence of hemolysis and catalase, lipase, phosphatase and protease activity; and susceptibility to *B. anthracis*–specific γ phage.

### DNA Preparation

*B. anthracis* cultures from the Kazakhstan National Collection were grown on Hottinger blood agar. A colony from each sample was harvested from the agar plates and dispersed in Tris-EDTA buffer for DNA extraction. A QIAamp DNA Mini Kit (QIAGEN, Valencia, CA, USA) was used to extract genomic and plasmid DNA by using the manufacturer’s protocol. A total of 1.0 mL of DNA was collected from each of the isolates in the collection.

### MLVA Genotyping

Eight VNTR (MLVA-8) markers were amplified by PCR by using primer pairs *vrrA*-f1 and *vrrA*-r1, *vrrB*_1_-f1 and *vrrB*_1_-r1, *vrrB*_2_-f1 and *vrrB*_2_-r1, *vrrC*_1_-f1 and *vrrC*_1_-r1, *vrrC*_2_-f1 and *vrrC*_2_-r1, CG3-f1 and CG3-r1, pXO1-AAT-f3 and pXO1-AAT-r3, and pXO2-AT-f1 and pXO2-AT-r1 (*4*)*.* One microliter containing ≈1 ng of template DNA was added to each PCR.

Electrophoresis of amplified products was performed on an ABI 310 genetic analyzer (Applied Biosystems, Inc., Foster City, CA, USA). Data were analyzed by using GeneMapper software V4.0 (Applied Biosystems, Inc.). To ensure comparability and accuracy of raw VNTR scores from the strains from Kazakhstan with the genotypes published by Keim et al. 2000 (*4*)*,* we performed electrophoresis on amplified fragments from 4 control DNAs (A0462-Ames, A0488-Vollum; A0071-Western North America and A0402; and French B2) in parallel with the isolates from Kazakhstan. In addition, DNA molecular size reference markers (Applied Biosystems, Inc) were included in each sample to accurately size the 8 VNTR fragments. Raw VNTR sizes were normalized to the sizes reported by Keim et al., 2000 (*4*) for genotypic comparisons.

### Unweighted Pair Group Method with Arithmetic Mean Cluster Analysis of Genotypes

Unweighted pair group method with arithmetic mean (UPGMA) cluster analysis of VNTR data from 92 confirmed *B. anthracis* isolates and the diverse 89 genotypes described by Keim et al. 2000 (*4*) were used to establish genetic relationships. Distance matrices were generated in PAUP 4.0 (Sinauer Associates, Inc., Sunderland, MA, USA) and imported into MEGA 3.1 (*13*) for tree-building purposes.

### Spatial Patterns of Genetic Relationships

The strain database was constructed from museum records and contemporary epidemiologic investigations. This database was synchronized with the bacterial culture collection to geolocate the culture by using the GIS. To map strain diversity, we categorized culture collection locations by strain identifications based on the MLVA genotyping results.

### SNP Typing of *B. anthracis* Isolates

Representative cultures from each Kazakh MLVA genotype plus the STI vaccine strain from Russia were genotyped by using previously described canonical SNPs discovered by whole-genome sequencing (*10**,**14*)*.* SNPs were interrogated by using the Roche Light Cycler II real-time PCR instrument (Roche Diagnostics, Indianapolis, IN, USA). Allelic discrimination assays initially developed on the ABI 7900 real-time platform (*10*) were adapted for use on the Light Cycler II. The assay amplifies a fragment of DNA sequence containing the SNP site. Two probes complementing the 2 potential SNP states were used as real time markers. Each probe had a distinct fluorescent label; i.e., probe 1 was labeled with 6-carboxy-fluorescein, and the alternate probe was labeled with VIC (Applied Biosystems, Inc.). The probe complementary to the sequence in the sample amplicon will hybridize over the SNP and surrounding sequence during the amplification process to generate a signal. It is possible for the incorrect probe to generate some signal but not enough to be confused as a positive reaction. The Light Cycler II discriminated which probe was the complementary sequence on the basis of the differential intensity of the reaction. Controls for each run included template DNA with both SNP states of interest.

## Results

### Historical Incidence and Geographic Distribution of Anthrax in Kazakhstan

**Figure 1:**
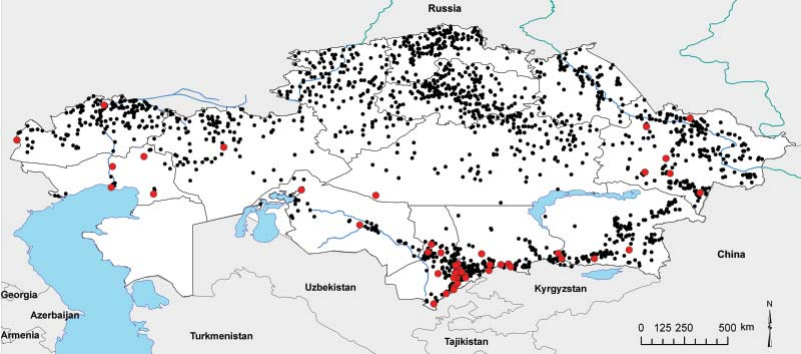
Anthrax outbreaks in Kazakhstan, 1937–2005. Each dot represents an outbreak; red dots indicate that cultures were isolated and analyzed from these outbreaks.

**Figure 2:**
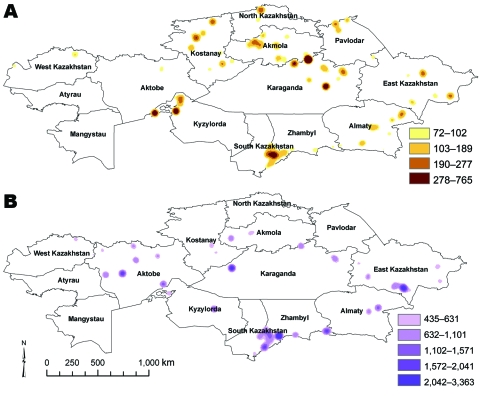
Kernel density estimates of anthrax outbreaks in cattle (A) and sheep (B), Kazakhstan, 1937–2005. Color shading represents SD values relative to density values from the kernel density estimate analysis for each species.

A total of 1,037 human outbreaks were reported, representing 1,765 human cases. The outbreaks occurred in 665 locations; 198 of those locations reported repeat outbreaks throughout the study period (Figure 1; Table 1). Additional review of historical data at the Kazakhstan Scientific Center for Quarantine and Zoonotic Diseases identified 3,947 outbreak events reported for animal species and were entered into GIS. The outbreaks occurred over 1,790 locations; 805 of those reported repeated outbreaks. Cattle and sheep were the primary livestock species affected during the study period; fewer outbreaks occurred among swine, and rarer, sporadic outbreaks occurred on mink farms and among foxes, and camels (Table 2). Cattle outbreaks were most common in northern Kazakhstan; several outbreaks occurred in the southernmost oblasts bordering Uzbekistan and Kyrgyzstan (Figure 2, panel A). Sheep outbreaks were prominent throughout eastern and southern Kazakhstan (Figure 2, panel B). The largest cattle outbreak (n = 174 cattle) in the dataset occurred in 1957 in the northernmost region of the Karaganda oblast in north central Kazakhstan. The largest sheep outbreak affected 851 sheep and occurred in the southern oblast of Zhambyl in 1971.

**Table 1:** Outcomes for 1,765 human patients in mapped anthrax outbreak areas, Kazakhstan, 1937–2005

Status	Number
Recovered	1,541
Deceased	75
Lost contact	17
No data/unknown	132

**Table 2:** Anthrax outbreaks, number of animal deaths per outbreak by species affected, and miscellaneous anthrax-positive samples, Kazakhstan, 1937–2005

Animal species	No. outbreaks/ samples	Deaths/ outbreak*	Total no. deaths
Sheep	1,735	0–851	16,080
Cattle	1,678	0–84	3825
Equine	304	0–28	634
Swine	192	0–78	832
Camel	5	1–2	7
Mink	3	28–37	95
Goat	1	1	1
Fox	1	1	1
Dog	2	1	2
Arctic fox	2	5	6
Unidentified	6	–	15
Miscellaneous anthrax-positive samples†			
Soil samples	17	–	–
Wool	1	–	–

*0 indicates animals that recovered from infection. †*Bacillus anthracis* spores were recovered, but there were no infections.

### Biochemical Tests

All cultures except 1 (isolate no. 49) were biochemically and morphologically consistent for *B. anthracis*; 3 cultures (isolate nos. 65, 76, and 77) were consistent with *B. anthracis* but did not exhibit capsule formation. With the exception of culture no. 49, isolates were nonhemolytic; nonmotile; phosphatase and lecithinase negative; protease, oxidase, and catalase positive; and, with 3 exceptions, formed a capsule.

### MLVA Genotyping

Of the 92 *B. anthracis* isolates, 88 isolates yielded complete data for the 8 marker MLVA; 3 isolates were missing the pX02 marker (isolate nos. 65, 76, and 77), and 1 was missing the pX01 plasmid marker (isolate no. 7). After we coded the raw VNTR fragment sizes, the Kazakh *B. anthracis* genotypes were analyzed by using PAUP 4.0 and MEGA 3.1 phylogenetic software programs. UPGMA cluster analysis of the Kazakh isolates with complete MLVA-8 data (*4*) identified 12 unique MLVA subtypes.

UPGMA cluster analysis of the 12 Kazak MLVA genotypes (G_kz_) with the diverse 89 genotypes reported by Keim et al. (*4*) showed that most isolates (n = 78) belonged to the previously described A1.a genetic cluster; 6 isolates belonged to the A3.b cluster; and 2 isolates belonged to the A4 cluster. More than half of the A1.a isolates belong to previously described genotypes (38/74; excluding samples with missing pX01, pX02 data), including the previously described MLVA genotypes 3 (n = 15), 6 (n = 2) and 13 (n = 21). Most of the novel genotypes reported from the Kazakhstan National collection represent slight variants of previously described genotypes that can be explained by the insertion or deletion of >1 tandem repeats at a particular locus, usually in pX01 or pX02 (Table 3). However, 2 of the genotypes from Kazakhstan (G_kz_-9 and -11) appear to represent new sublineages on the basis of newly described allele combinations and distance-based clustering with the diverse 89 genotypes. In addition, the pX01 allele sizing at position 138 appears novel (G_kz_-5); we have not seen this size reported in previous MLVA-8 studies (Table 3).

**Table 3:** Variable number tandem repeat sizes for *Bacillus anthracis* isolates, Kazakhstan*

Kazakhstan genotype no.	MLVA group†	MLVA genotype	*vrrA*	*vrrB1*	*vrrB2*	*vrrC1*	*vrrC2*	*CG-3*	*pX01*	*pX02*
1	A1.a	Gt-13 (*4*)	313	229	162	613	604	153	132	137
2	A1.a	Novel	313	229	162	613	604	153	135	137
3	A1.a	Novel	313	229	162	613	604	153	129	139
4	A1.a	Novel	313	229	162	613	604	153	129	137
5	A1.a	Novel	313	229	162	613	604	153	138	137
6	A1.a	Gt-6 (*4*)	301	229	162	613	604	153	126	137
7	A1.a	Gt-3 (*4*)	313	229	162	613	604	153	126	137
8	A1.a	Novel	313	229	162	613	604	153	132	139
9	Novel	Novel	325	229	162	613	604	158	132	137
10	A4	Novel	313	229	162	538	604	158	126	137
11	Novel	Novel	313	229	162	583	532	153	129	141
12	A3b	Novel	313	229	162	583	532	158	126	139

*Raw allele sizes were determined by electrophoresis on the ABI 310 (Applied Biosystems, Inc., Foster City, CA, USA); sizes were compared to control variable number tandem repeats and corrected to the sizes reported by Keim et al. (*4*). †MLVA, multilocus variable number tandem repeat. MLVA group determined by unweighted pair group method arithmetic mean clustering with the diverse 89 genotypes described by Keim et al. (*4*).

### Geographic Distribution of MLVA Genotypes

**Figure 3:**
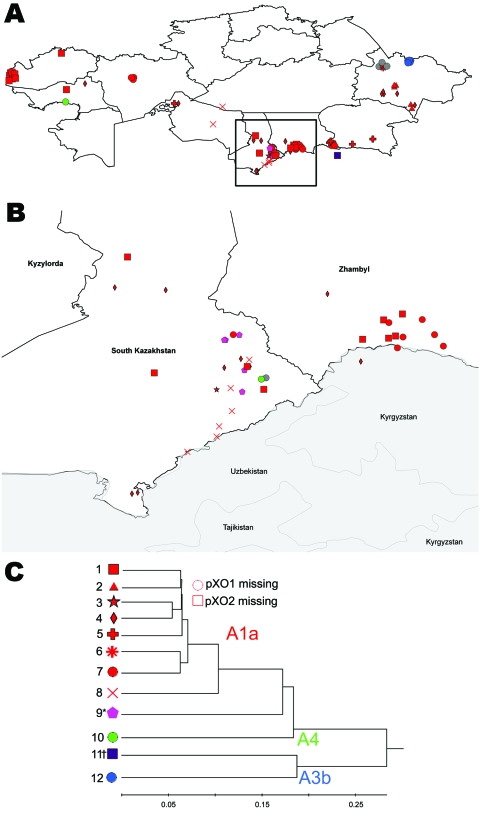
Geographic distribution of genotypes of *Bacillus anthracis* strains in Kazakhstan (A), with a closer view of outbreaks within eastern and southern Kazakhstan (B). Different genotypes are represented by different shapes and color coding reflecting major genetic affiliations (C). * and † indicate novel subgroups. Scale bar indicates genetic difference.

The geographic distribution of MLVA types in Kazakhstan indicated that A1.a genotypes were widely distributed (Figure 3). For example, the most common Kazakh genotype (G_kz_-1; n = 21) clusters on the Georgia–Kazakhstan border and on the southern border near Kyrgyzstan and Uzbekistan. The A1.a G_kz_-4 (n = 17) is also widely dispersed across Kazakhstan; cases have occurred in the western, southern, and eastern regions and into Kyrgyzstan. Specific genotypes within the Kazakh A1.a group appear to exhibit geographic clustering, reflecting temporally linked outbreaks.

The KZ genotypes 9–12 (G_kz_-9–12) also appear to be more geographically confined, although this apparent confinement is likely a reflection of sample size. For example, isolates with G_kz_-12 (n = 6; Figure 3) are exclusively found in the border region of the East Kazakhstan oblast, whereas the group 9 isolates (n = 5) are found in the Shymkent oblast in the south-central portion of the country. MLVA G_kz_-11 (n = 1), which appears to represent a previously unreported genetic lineage, was isolated just south of Kazakhstan in Kyrgyzstan.

### SNP Typing

Representative cultures from each of the Kazakh MLVA genotypes plus the Russian STI vaccine were SNP genotyped by using allelic discrimination probes and the Light Cycler II instrument. The SNP results were compared (Table 4) with the SNP profiles of Van Ert et al. (*10*), allowing assignment of the isolates to 1 of 12 sublineages. As with MLVA typing, all isolates tested with SNPs had genotypes characteristic of the A branches.

**Table 4:** *Bacillus anthracis* SNPs, Kazakhstan*

Isolate	KZ MLVA genotype	SNP group	SNPs												
			A branch									B branch			
			001	002	003	004	006	007	008	009		001	002	003	004
KZ 6	1	A.Br.008/009	T	G	A	T	A	T	G	A		T	G	G	T
KZ 60	2	A.Br.008/009	T	G	A	T	A	T	G	A		T	G	G	T
KZ 52	3	A.Br.008/009	T	G	A	T	A	T	G	A		T	G	G	T
KZ 3	4	A.Br.008/009	T	G	A	T	A	T	G	A		T	G	G	T
KZ 44	4	A.Br.008/009	T	G	A	T	A	T	G	A		T	G	G	T
KZ 1	5	A.Br.008/009	T	G	A	T	A	T	G	A		T	G	G	T
KZ 74	6	A.Br.008/009	T	G	A	T	A	T	G	A		T	G	G	T
KZ 25	7	A.Br.008/009	T	G	A	T	A	T	G	A		T	G	G	T
KZ 55	7	A.Br.008/009	T	G	A	T	A	T	G	A		T	G	G	T
KZ 8	8	A.Br.008/009	T	G	A	T	A	T	G	A		T	G	G	T
KZ 13	9	A.Br.008/009	T	G	A	T	A	T	G	A		T	G	G	T
KZ 11	10	A.Br.Vollum	T	G	A	T	A	C	T	A		T	G	G	T
KZ 42	11	A.Br.Ames	C	A	G	C	A	T	T	A		T	G	G	T
KZ 66	12	A.Br.Ames	C	A	G	C	A	T	T	A		T	G	G	T
KZ ST1	NA	A.Br.008/009	T	G	A	T	A	T	G	A		T	G	G	T

*SNP, single nucleotide polymorphism; KZ, Kazakhstan; MLVA, multilocus variable number tandem repeats; NA, not applicable. SNP changes are shaded. SNP groups as described in Van Ert et al. (*10*).

Representatives of MVLA genotypes 1–9 were assigned to A.Br.008/009, KZ genotype 10 to the A.Br.Vollum subgroup, and genotype 11 and 12 to the A.Br.Ames subgroup. The SNP data indicated that all representative A1.a Kazakh isolates belonged to the European branch of this group. The assignment of MLVA G_kz_-10 to the A.Br.Vollum group is consistent with *B. anthracis* found globally in areas such as Pakistan and western China (*10*). Likewise, the assignment of Kazakh MLVA genotypes 11 and 12 to the A.Br.Ames genotype is consistent with the presence of this lineage in China (*10*)*.*

## Discussion

The historical occurrence and geographic distribution of anthrax outbreaks in Kazakhstan suggest anthrax foci are heavily concentrated in the southern region and broadly distributed across the northern portions of the country but are less common in the central regions. This may reflect regional differences in soil composition, availability of water and livestock and even case reporting. For example, the central region of Kazakhstan is dominated by desert, which likely has poor soils for long-term spore survival, whereas in the southern, northern, and eastern oblasts, the soils are more alkaline with higher organic matter and likely support spore survival (*15**–**17*)*.* From a temporal perspective, outbreaks (or outbreak reports) have decreased in severity (number of animals infected), frequency (number of reported outbreaks), and have been associated with smaller geographic areas affected. However, the spatial distribution of the disease appeared to be relatively stable in the northern and southern Kazakh oblasts during the study period.

From a genetic perspective, *B. anthracis* in Kazakhstan was dominated by isolates clustering in the MLVA A1.a group, which is consistent with reports of the A1.a group being widely distributed globally (*4**,**5**,**6*)*.* The widespread occurrence and apparent ecologic establishment of these VNTR genotypes in Kazakhstan supports the hypothesis that the A1.a group represents a very fit strain complex (*6*)*.* Of the 8 A1.a genotypes in Kazakhstan, 5 were novel (G_kz_-2, -3, -4, -5, and -8) and exhibited a previously undescribed pX01 allele (G_kz_-5), which is not unexpected considering that this region has been underrepresented in prior MLVA-8 *B. anthracis* studies (*4**–**8*).

SNP typing of representative isolates from the A1.a Kazakh MLVA genotypes assigns these isolates to the A.Br.008/009 SNP lineage, which is widely distributed throughout Europe and has been reported in western China (*10*,*18*). Notably, the SNP data differentiate the Kazakh genotypes from the related North American genotypes, which are not effectively differentiated by MLVA alone. Since the representative Kazakh isolates in this SNP study were cultured from outbreaks spanning a 50-year period (1952–2002), our data not only expand the understanding of the geographic range of this Eurasian lineage (A.Br.008/009) but also provide insights into its historical incidence and persistence in the country. Because of sampling limitations, the extent to which this dominant lineage is represented in the northern sections of Kazakhstan, and further into Russia, is unknown. However, in a recent study *B. anthracis* DNA from persons affected by the Sverdlovsk accident was assigned to the A.Br.008/009 SNP subgroup (*19*). Our data and the report that the Sverdlovsk strain was initially isolated in the 1950s in Kirov, Russia (*19*), underscores the need to genotype additional samples in northern Kazakhstan oblasts and Russia to measure the northern range of this apparently highly successful lineage.

The assignment of Kazakh isolates to the A3.b and A4 MLVA clades and the A.Br.Ames and A.Br.Vollum SNP groups is not surprising considering these MLVA and SNP types are also found in Middle Eastern countries, such as Pakistan and China (*10*)*.* As first reported by Van Ert et al. (*10*), and later detailed by Simonson et al. (*18*), the A.Br.001/002 is common in China, whereas the closely related A.Br.Ames SNP lineage is more restricted geographically. The finding that the Kazakh isolates from the eastern border were assigned to A.Br.Ames SNP group is notable considering that the A.Br.Ames isolates that can be geolocated are found exclusively in Inner Mongolia. These genotypic similarities may reflect historical trade and nomadic routes linking those regions.

The absence of B lineage genotypes in Kazakhstan, as indicated by both MLVA and SNP data, is consistent with the lack of these genotypes in China, including the western province of Xinjiang (*10*,*18*), and supports the hypothesis that these lineages are restricted to narrow environmental conditions and, therefore, are more restricted in their global distribution (*9*)*.* On a more local level, our MLVA data permit strain-level analysis of samples isolated during outbreaks. In several instances we were able to link strains collected from human anthrax patients to the infection source. For example, we identified the same strain in 10 cultures collected from an outbreak in western Kazakhstan that occurred from July–August 2005. The samples included cultures isolated from livestock, contaminated meat, human victims, and contaminated soil. The MLVA data linked the cultures and provided a mechanism for retrospective epidemiologic trace-back.

Sampling biases and limitations are important considerations in any study. For example, the distribution of cultures available for this study does not represent a balanced sampling of the entire country. There is an ongoing effort in Kazakhstan to expand the culture collection and to include a wider geographic sampling of the country, including the northern oblasts, which is underrepresented in the current culture collection but has a long historical record of anthrax. It would be worthwhile to revisit livestock burial sites and to isolate and analyze cultures from this region. In addition, the application of more comprehensive genetic analysis of Kazakh isolates would provide greater insight into the uniqueness of *B. anthracis* diversity in this region. For example, although canonical SNPs provide a powerful tool for assigning isolates into major clonal lineages, their resolution is limited by the use of relatively few representative SNPs and the diversity of the genomes used in the initial discovery process.

In summary, our work describes the historical incidence, distribution, and biochemical and genetic diversity of *B. anthracis* isolates in the central Asian republic of Kazakhstan. Our discovery of novel genotypes in this region contributes to the understanding of the global diversity of the pathogen and emphasizes the need for future studies in this geographic region. In addition, this study provides useful baseline data for future epidemiologic studies in Kazakhstan and for guiding future disease control programs
